# iNKT Cells and Their Potential Lipid Ligands during Viral Infection

**DOI:** 10.3389/fimmu.2015.00378

**Published:** 2015-07-24

**Authors:** Anunya Opasawatchai, Ponpan Matangkasombut

**Affiliations:** ^1^Department of Microbiology, Faculty of Science, Mahidol University, Bangkok, Thailand; ^2^Faculty of Dentistry, Mahidol University, Bangkok, Thailand; ^3^Systems Biology of Diseases Research Unit, Faculty of Science, Mahidol University, Bangkok, Thailand

**Keywords:** iNKT, CD1d, virus, lipid

## Abstract

Invariant natural killer T (iNKT) cells are a unique population of lipid-reactive CD1d-restricted innate-like T lymphocytes. Despite being a minor population, they serve as an early source of cytokines and promote immunological crosstalk thus bridging innate and adaptive immunity. Diseases ranging from allergy, autoimmunity, and cancer, as well as infectious diseases, including viral infection, have been reported to be influenced by iNKT cells. However, it remains unclear how iNKT cells are activated during viral infection, as virus-derived lipid antigens have not been reported. Cytokines may activate iNKT cells during infections from influenza and murine cytomegalovirus, although CD1d-dependent activation is evident in other viral infections. Several viruses, such as dengue virus, induce CD1d upregulation, which correlates with iNKT cell activation. In contrast, herpes simplex virus type 1 (HSV-1), human immunodeficiency virus (HIV), Epstein–Barr virus, and human papilloma virus promote CD1d downregulation as a strategy to evade iNKT cell recognition. These observations suggest the participation of a CD1d-dependent process in the activation of iNKT cells in response to viral infection. Endogenous lipid ligands, including phospholipids as well as glycosphingolipids, such as glucosylceramide, have been proposed to mediate iNKT cell activation. Pro-inflammatory signals produced during viral infection may stimulate iNKT cells through enhanced CD1d-dependent endogenous lipid presentation. Furthermore, viral infection may alter lipid composition and inhibit endogenous lipid degradation. Recent advances in this field are reviewed.

## Introduction

Since they were first described in the late 1980s, invariant natural killer T (iNKT) cells have been recognized as a minor, but unique lipid-reactive population of T cells with diverse functions in the immune system. The number of iNKT cells in human peripheral blood is highly variable and ranges from 0.03 to 0.78% of lymphocytes (1). They function similarly to the cells of the innate immune system as they display less specificity and more rapid activation compared to adaptive immune cells (2). The term “invariant” comes from the expression of almost invariant T cell receptors (TCR), Vα14 Jα18 in mice and Vα24 Jα18 in human, paired with limited Vβ chain (3). Unlike conventional T cells, which recognize peptide antigens presented on MHC molecules, iNKT cells recognize lipid antigens presented on CD1d. A member of the CD1 family, CD1d is a non-polymorphic MHC class I-like molecule, expressed on antigen-presenting cells (APCs). CD1d is present on dendritic cells (DC), B cells, monocytes, and macrophages and also on cells of non-hematopoietic origin, such as lung, gastrointestinal and cervical epithelial cells, and hepatocytes (4, 5).

Although not the main focus of this review, it should be noted that in addition to iNKT or type 1 NKT cells, there is another NKT cell population called diverse NKT (dNKT) or type 2 NKT cells. The dNKT cells express TCRs that are more diverse and recognize different sets of lipid antigens compared to iNKT cells (6). Furthermore, other CD1 family members, such as CD1a, b, c, present other types of lipid structures and are able to activate CD1-reactive, non-NKT T cells, such as γδT cells (7).

Activated iNKT cells can rapidly produce various T helper cell cytokines and crosstalk with other populations of cells in the immune system. Thus, they are an important factor in determining the outcome of the overall immune responses in various disease models, such as asthma (8), autoimmune diseases (9), cancer (10), and infectious diseases (11). The diverse roles of iNKT cells from anti-microbial immunity to regulatory functions in autoimmune diseases are partly due to the bidirectional activation between iNKT cells and DCs. In the presence of infection or pattern recognition receptor stimulation, iNKT–DC interactions, through CD40–CD40L, induce NF-kB activation, enhancing pro-inflammatory DC maturation and IL-12 production (12). Simultaneously, DCs present lipid on CD1d and produce IL-12 activating iNKT cells. The activation of these pathways results in the induction of innate and adaptive immune responses, including transactivation of NK cells (13) and enhanced response of CD4^+^ and CD8^+^ classical T cells to peptide antigens (2, 14, 15). In contrast, interactions between iNKT cells and immature DC, without other stimuli, trigger tolerogenic DC maturation. Tolerogenic DCs in turn induce regulatory T cells preventing autoimmunity (12).

Invariant natural killer T cells can be activated directly by the cognate interactions between their invariant TCRs and CD1d-loaded with exogenous or endogenous lipid antigen, and indirectly by the combination of pro-inflammatory cytokines (11). The first identified exogenous lipid antigen for iNKT cells was α-galactosylceramide (α-GalCer), a glycosphingolipid. Subsequently, pathogenic bacteria-derived glycolipids from *Borrelia burgdorferi* (16) and *Streptococcus pneumoniae* (17) were found to bind CD1d and be presented to iNKT cells. In the absence of microbial-derived or exogenous lipid antigens, such as in the case of Gram-negative *Salmonella* infection (18, 19), iNKT cell activation can also be mediated by presentation of endogenous lipid antigens via cognate interaction between CD1d and iNKT cell TCR, as well as cytokine-mediated activation (11).

Viruses are another example of microbes that lack lipid antigens, yet there is growing evidence for the involvement of iNKT cells in several viral infections (20). The mechanisms underlying iNKT cell activation during viral infection remain ambiguous. While some studies suggest cytokine-mediated activation, others indicate possible lipid-loaded CD1d-dependent activation. Several lines of study have clearly demonstrated that some viruses downregulate surface CD1d expression, attenuating the iNKT cell response as an evasion strategy, supporting a role for CD1d-dependent iNKT cell activation in viral clearance (21–25).

In this review, we summarize the current information on the role of iNKT cells, CD1d, and lipid antigens during viral infection. Importantly, potential CD1d-loaded lipid antigens as iNKT cell ligands in viral infection will be discussed and proposed.

## iNKT Cells in Viral Infection

Both protective and pathogenic roles of iNKT cells in various viral infections have been demonstrated in mice and human. Mice lacking iNKT cells displayed worsened disease outcomes for several viral infections including herpes simplex virus type 1 and 2 (HSV-1, 2) (24, 26, 27), murine cytomegalovirus (MCMV) (28), respiratory syncytial virus (RSV) (29), and influenza virus (30–32). In human, human immunodeficiency virus (HIV) is known to infect CD1d-restricted T cells (33), resulting in reduced iNKT cell numbers in HIV-infected patients after seroconversion (34). Moreover, X-linked lymphoproliferative syndrome patients, who have mutations in SLAM-associated protein, an adaptor protein important for iNKT cell development, are more susceptible to severe Epstein–Barr virus (EBV) infection suggesting a protective role for iNKT cells against EBV infection (35–37).

Beneficial roles of iNKT cells are also demonstrated by the enhanced anti-viral immunity and improved clinical outcomes following treatment with α-GalCer, a potent iNKT cell stimulant, in HIV (38), MCMV (39), RSV (29), hepatitis B virus (HBV) (40), and influenza virus infections (41). Co-administration of α-GalCer with inactivated influenza virus resulted in boosted antibody production and enhanced cellular responses to subsequent infections in immunized mice (42). In contrast, iNKT cells are also known to have pathogenic roles following hepatitis C virus (HCV) infection (43), and promote chronic lung disease in Sendai virus-infected mice (44). Recently, iNKT cells have been shown to play a deleterious role in dengue virus (DENV) infection in mice (45), and iNKT cell activation was found to be correlated with poor clinical outcomes in dengue infected patients (46).

## Modes of iNKT Cell Activation during Viral Infection

As viruses contain no known exogenous lipid antigens, it is possible that they may activate iNKT cells using cytokine signals alone or through CD1d-bound endogenous lipid antigens. For some viruses, such as influenza (31) and MCMV (47), cytokines secreted during infection alone could potentially activate iNKT cells. While the significance of CD1d-dependent iNKT cell activation in viral infection remains controversial, APC stimulation by viral toll-like receptor (TLR) agonists has been shown to lead to a shift in cellular lipid metabolism toward antigenic lipids as well as CD1d-dependent iNKT cell activation (48, 49). Moreover, some viruses downregulate CD1d expression, presumably to evade iNKT cell recognition, suggesting that CD1d-bound endogenous lipid antigens might be involved in iNKT cell response during viral infection. Because dNKT cells are also reactive to CD1d-loaded lipids, the up- or downregulation of CD1d in viral infection could also affect dNKT cells. Likewise, the expression of different CD1 isoforms could also affect the functions of other CD1-reactive T cells such as γδT cells.

## Regulation of CD1d in Viral Infection

### CD1d upregulation

CD1d expression is upregulated in response to viral danger signals, and the increase in expression could lead to higher iNKT cell response (23, 50). DC maturation in response to viral TLR agonists leads to higher cell surface CD1d expression (49). The increase in cell surface CD1d in response to viral TLR agonists was shown to be mediated both at the transcriptional level and through enhanced cellular distribution of CD1d toward the surface (50). Apart from viral TLR stimulation, type I interferons, known for their anti-viral function, can also induce higher levels of CD1d mRNA transcripts (50). In actual viral infections, CD1d was upregulated in cardiac endothelial cells in mice infected with coxsackievirus B3 virus (51), hepatocytes from HCV infected patients (52), and in monocytes from DENV-infected patients (46).

The upregulation of CD1d in response to viral danger signals could therefore be a possible mechanism for initiating the iNKT cell response to the viral infection. This notion has been supported by experiments demonstrating that iNKT cell cytokine production in response to attenuated HSV was reduced upon blocking CD1d with a monoclonal antibody (50). In addition, induction of CD1d expression in EBV-transformed B cells has also been shown to rescue IFN-γ production in iNKT cells (23). However, blocking of CD1d through the use of an antibody in attenuated HSV-infected DC could not completely abrogate the iNKT cell response (50). Moreover, induction of CD1d expression in healthy B cells did not result in an enhanced iNKT cell response (23). These findings suggest that additional soluble factors, such as cytokines produced during viral infection, might act in concert with CD1d antigen presentation to optimize the iNKT cell response.

### CD1d downregulation: A strategy to subvert iNKT cell recognition?

Another piece of evidence supporting a CD1d-dependent response during viral infection is the finding that some viruses downregulate surface CD1d. This ability has been hypothesized to be a strategy to subvert iNKT cell recognition. The earliest reports of viral-infection-induced CD1d downregulation were from lymphocytic choriomeningitis virus (LCMV), vaccinia virus (VV), and vesicular stomatitis virus (VSV) (53) infections. Mice with acute LCMV, VV, and VSV infections showed reduced surface CD1d expression on DCs and macrophages (53). A subsequent analysis demonstrated that the VSV protein could affect cellular CD1d distribution resulting in inhibition of CD1d-mediated antigen presentation (54). HSV-1 (21, 55–57) and Kaposi sarcoma-associated herpes virus (KSHV) (25) also utilize their viral proteins to disturb CD1d trafficking, in these cases, through interaction with the CD1d cytoplasmic tail, a site important for CD1d sorting. While HSV viral proteins modify and signal CD1d for lysosomal degradation (57), interaction with KSHV proteins increases CD1d internalization from the cell surface (25). EBV, another member of herpes viruses, has recently been shown to downregulate CD1d expression on EBV-transformed B cells abrogating the recognition by iNKT cells (23). In contrast to HSV and KSHV, the decrease in CD1d expression during EBV infection is a result of altered CD1d transcription (23). Human papillomavirus (HPV) employs yet another strategy to suppress surface CD1d expression, utilizing a viral protein E5 to trap CD1d molecules inside the ER-promoting proteasomal degradation (58). Three different HIV proteins VpU (59), Nef (22, 60), and gp120 (61) participate in CD1d downregulation, but whether CD1d downregulation results in the loss of iNKT cell recognition in HIV-infected patients is still unknown.

**Figure 1:**
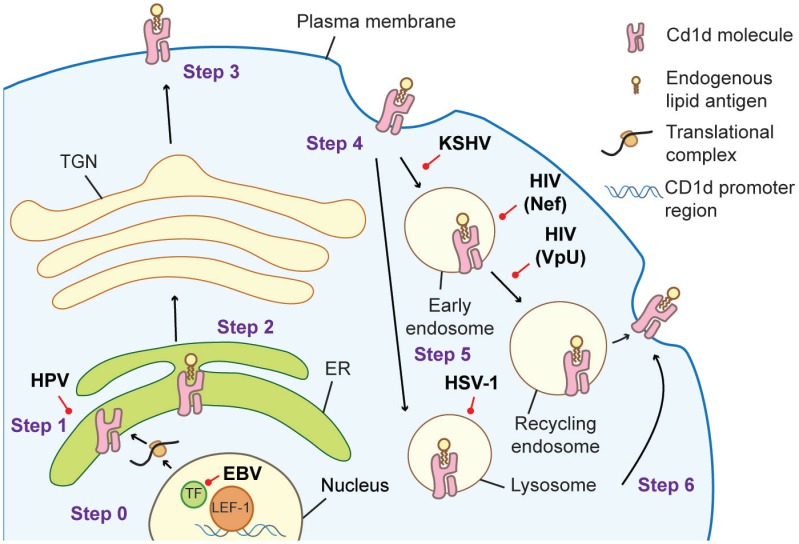
**Cellular trafficking of CD1d molecules in an antigen-presenting cell (APC) and the strategies viruses employed to interfere with successful antigen presentation to iNKT cells**. Step 0: CD1d gene is transcribed. EBV infection results in the association of LEF-1 at the CD1d promoter region interfering with its transcription (23). Step 1: newly synthesized CD1d molecules assemble with the β_2_-microglobulin subunit in the endoplasmic reticulum (ER). HPV utilizes its E5 protein to retain CD1d in the ER (58). Step 2: endogenous lipid antigen is loaded on CD1d. Step 3: loaded CD1d traffics to the plasma membrane. Step 4: CD1d is internalized into the endocytic compartments. The MIR protein of KSHV can promote endocytosis (25). The HIV protein Nef accelerates CD1d internalization (22, 60), while VpU retains it in early endosome (59). HSV-1 infection results in CD1d retention in the lysosomal limiting membrane (21), and two HSV-1 proteins gB and US3 direct CD1d to lysosomal degradation (55). Step 5: the exchange for the antigenic lipid occurs in the lysosome. Step 6: CD1d returns to the plasma membrane to present lipid antigen to iNKT cell membrane to present lipid antigen to iNKT cells.

These examples highlight several strategies employed by viruses to achieve one goal, to prevent CD1d from reaching or accumulating at the cell surface (Figure 1). The downregulation of CD1d can diminish the iNKT cell response and worsen the outcome of several viral infections, suggesting that iNKT cells might be a significant player in combating against certain viral infections. Together, these findings demonstrate the importance of CD1d-dependent iNKT cell activation in the cellular response to viral infection even though viruses contain no known exogenous lipid antigens. The next challenge in understanding how iNKT cells are modulated by viral infections is the identification of potential endogenous lipid antigens that could serve as iNKT cell ligands.

## Possible Self-Lipid Ligands for iNKT Cells

Due to the lack of virus-derived lipid antigens, host cellular lipids are the most likely source of CD1d ligands that are presented to activate iNKT cells during viral infection. Endogenous lipid antigens are required for iNKT cell selection in the thymus and possibly play a role in activating iNKT cells in the periphery (3). The advancement in the search for endogenous lipid ligands has begun to provide insights into the biology of CD1d-bound mammalian lipids that could induce the iNKT cell response. However, the role of endogenous lipids as well as their regulation during viral infection remains largely unknown.

### Cellular lipid antigens

#### Glycosphingolipids

Several lines of evidence have suggested a role for mammalian glycosphingolipids (GSLs) in the development and peripheral activation of iNKT cells. Among these GSLs, isoglobotrihexosylceramide (iGb3) was proposed to be involved in thymic iNKT cell selection and peripheral iNKT cell activation (18, 62). However, its importance in these processes remains to be clarified (63, 64).

Glucosylceramide (GlcCer) derivatives can initiate an iNKT cell response in CD1d-dependent manner (48, 49, 65). β-anomeric GSLs were previously considered as the candidate endogenous iNKT ligands as these lipids are the most abundant form of GSLs in mammalian tissues. In addition, only β-transferases for GlcCer and galactosylceramide are present in the mammalian genome (66) and α-anomeric GSLs were not thought to be present in mammals (67). However, recent findings, using more sensitive lipid detection methods, suggest that α-anomeric GSLs might be sparingly present in the mammalian cells (66, 68). Interestingly, the activity of GlcCer from mammalian tissue, formerly ascribe as β-GlcCer (48), is now found to account for a low level of α-anomeric GSL that appears to impact iNKT activity. Removal of β-GlcCer from the lipid fraction using glucocerebrosidase treatment did not alter iNKT cell activity while inhibition of α-anomeric GSL with a monoclonal antibody diminished the effect (66, 68). In addition, only α-GalCer but not β-GluCer-loaded CD1d tetramer could stain splenocytes and DN32 NKT hybridoma (66). Consistent with these observations, mice treated with an antibody against α-linked monoglycosylceramide exhibited impaired iNKT cell development (66). This suggests that α-linked monoglycosylceramides, such as α-GalCer and α-GlcCer, might be the iNKT cell selecting self-antigen in the thymus (66). The availability of α-GlcCer is tightly regulated by degradation with catabolic enzymes of the ceramide and glycolipid pathway (66). However, the detailed mechanisms underlying the synthesis of α-anomeric GSLs in mammals remain largely unknown.

#### Non-Glycosphingolipids

Apart from GSLs, other lipid species have also been suggested as possible iNKT cell stimuli (69). Phosphatidylinositol (PI) (70) and phosphatidylcholine (PC) (71) were among the first endogenous CD1d-bound lipids reported. Mammalian lysophospholipids and lysosphingomyelin stimulate iNKT cell hybridomas with varying strength among the clones examined (72). A recent study also demonstrated that ether-bonded phospholipids generated in the peroxisomes of mouse thymus could serve as iNKT cell selecting ligands, as mice lacking the enzyme required for their generation displayed a marked decreased in iNKT cell number (73).

### Regulation of endogenous lipid antigens presented in response to TLR stimulation and infection

Limited evidence is available regarding CD1d-dependent endogenous lipid presentation to iNKT cells during viral infection. By presenting self-lipid antigens, the host is at risk for undesirable auto-reactivity, necessitating tight control of this process. Unresolved questions regarding endogenous lipid antigens utilized in the response to viral infection include what are the correct form of lipid antigen, the appropriate magnitude of release, as well as location, and temporal control that would provide a beneficial effect but limit adverse consequences to the host. Several studies suggested the possible involvement of the pro-inflammatory signals that enhance CD1d-dependent self-lipid presentation (48, 49, 74), self-lipids “alteration” during viral infection (75), or the inhibition of enzymes that degrade endogenous lipid antigens (76).

**Figure 2:**
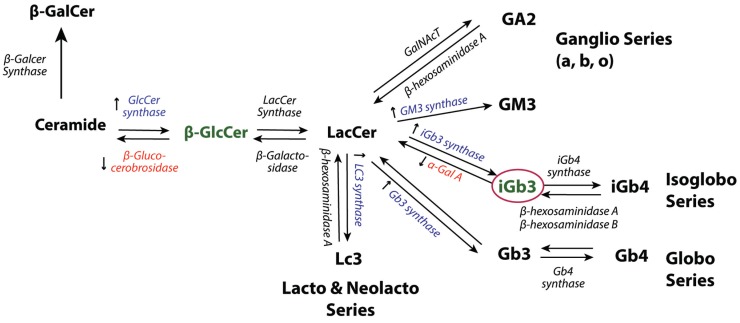
**GSL synthetic pathways**. Accumulation of GlcCer (48) and iGb3 (18, 76), two potential endogenous iNKT cell stimuli in response to infection or TLR stimulation. Consistent with the proposed self-lipids, ceramide glucosyltransferase (GlcCer synthase) (48, 49), GM3 synthase (49), iGb3 synthase (49), Gb3 synthase (49), and LC3 (49) are reported to be upregulated at the transcriptional level in response to TLR stimulation. Downregulation of β-glucocerobrosidase (48) and decreased activity of α-galactosidase A (α-Gal A) (76) have also been shown in response to TLR stimulation.

Several innate pro-inflammatory signals may induce iNKT cell activation through CD1d presentation of endogenous lipid antigens. Once DCs are stimulated with agonists for endosomal TLRs known to recognize viral genomes, such as TLR3 (77), TLR7 (74), and TLR9 (49, 78), they may mount an iNKT cell response by the presentation of endogenous lipid antigen in concert with the production of cytokines such as IL-12 (74, 77, 78) and type I interferon (49). Although several enzymes involved in the biosynthesis of GSLs including GlcCer synthase and sialyltransferases were found upregulated in response to TLR stimulation (Figure 2), the lipid antigen being presented remains elusive (49, 74). β-GlcCer was proposed to be the endogenous ligand involved in the iNKT cell response to TLR stimulation (48) but whether low levels of α-GlcCer contamination could be the active ligand in this situation was not examined (66, 68). Although α-linked monoglycosylceramide has been suggested as the selecting ligand for iNKT cell development (66), it is not known whether it plays a role in viral infection.

The alteration of self-lipid antigens to “antigenic” lipids that could activate CD1d-restricted NKT cells was reported in the mouse model of HBV infection (75). Hepatocytes infected with HBV could induce NKT cell activation in a process that required CD1d, a microsomal triglyceride transfer protein (MTP) and secretory phospholipases. The antigenic lipids were found to be lysophospholipids, specifically lysophosphatidylethanolamine (PE) (75). Surprisingly, *in vitro* analysis indicated that iNKT cells were not activated by CD1d-presented lysophospholipids, instead a dNKT cells appeared to be the target. Moreover, the activation of iNKT cells was shown to be cytokine mediated during *in vivo* murine HBV infection (75). Therefore, the nature of lipid antigens, differences in TCR structure, and the mode of docking of different lipid antigens might contribute to their activation efficacy on different NKT cell subsets.

Decreased degradation of endogenous lipid antigens has been suggested to mediate iNKT cell auto-reactivity and activation in response to TLR stimulation (76). The activation of MyD88-dependent-TLR 4 and 9 could lead to a decrease in the enzymatic activity of α-galactosidase A, an enzyme that acts as a rate limiting step in endogenous lipid degradation and results in the accumulation of lysosomal lipid and iNKT cells activation (76) (Figure 2). α-Gal-A is proposed to play a role in regulating the cellular levels of GSLs during physiologic conditions, but decreases in its activity may allow the level of self-lipid to reach the threshold of iNKT cell stimulation during infection (76).

## Alteration of Lipid Metabolisms during Viral Infection

Recent findings indicate that viruses can modulate host lipids to accommodate their life cycle. Several cellular lipids have been identified to be crucial for their entry, replication, and budding (79). Altering the host metabolism as a strategy to facilitate their replication has been reported for human cytomegalovirus (80), DENV (81), and HCV (82) infection. Two HCV proteins, NS5A and NS5B, appear to induce expression of the GlcCer synthase gene, an enzyme essential for the synthesis of GlcCer, a species of self-lipid antigen (83) (Figure 2). Whether such alterations by the virus to accommodate itself could serve as a signal for the immune response to counteract the infection is not clear. Likewise, whether changes in lipid metabolism mediated by virus infection can be employed as an immune evasion strategy by diverting the lipid profile “away” from the composition that could activate iNKT cells has not yet been extensively studied.

## Concluding Remarks

Despite the rapidly expanding knowledge regarding the roles of iNKT cells in viral infection, an important question remains: what are their natural ligands during viral infection? Self-lipid antigens loaded on CD1d have been proposed in the absence of microbial-derived lipid antigens. Recent advances using viral TLR agonists have identified potential species of cellular lipids as iNKT cell stimuli. However, iNKT cell lipid ligands important in actual viral infections have not been established. As several viruses are known to interfere with host cellular lipid metabolism, alteration of cellular lipid regulation may also affect self-lipid antigen presentation to iNKT cells. A better understanding of self-lipid antigens in viral infection would not only provide us with a more complete picture of the complex host–virus interaction but would also reveal potential strategies to manipulate iNKT cells for desirable effects to combat against viral infections.

## Conflict of Interest Statement

The authors declare that the research was conducted in the absence of any commercial or financial relationships that could be construed as a potential conflict of interest.
